# Transgenic overexpression of ITGB6 in intestinal epithelial cells exacerbates dextran sulfate sodium‐induced colitis in mice

**DOI:** 10.1111/jcmm.16297

**Published:** 2021-01-24

**Authors:** Haiyan Chen, Liubo Chen, Xin Wang, Xiaoxu Ge, Lifeng Sun, Zhanhuai Wang, Xiaoming Xu, Yongmao Song, Jing Chen, Qun Deng, Haiting Xie, Ting Chen, Yan Chen, Kefeng Ding, Jingjing Wu, Jian Wang

**Affiliations:** ^1^ Department of Colorectal Surgery and Oncology Key Laboratory of Cancer Prevention and Intervention Ministry of Education The Second Affiliated Hospital Zhejiang University School of Medicine Hangzhou China; ^2^ Department of Radiation Oncology Key Laboratory of Cancer Prevention and Intervention Ministry of Education The Second Affiliated Hospital Zhejiang University School of Medicine Hangzhou China; ^3^ Department of Pathology & Pathophysiology, and Department of Colorectal Surgery of the Second Affiliated Hospital Zhejiang University School of Medicine Hangzhou China; ^4^ Center for Inflammatory Bowel Diseases The Second Affiliated Hospital Zhejiang University School of Medicine Hangzhou China; ^5^ Department of Pathology The Second Affiliated Hospital Zhejiang University School of Medicine Hangzhou China; ^6^ Key Laboratory of Cancer Prevention and Intervention Ministry of Education The Second Affiliated Hospital Cancer Institute Zhejiang University School of Medicine Hangzhou China

**Keywords:** DSS‐induced colitis, IBD, integrin αvβ6

## Abstract

Integrins, as a large family of cell adhesion molecules, play a crucial role in maintaining intestinal homeostasis. In inflammatory bowel disease (IBD), homeostasis is disrupted. Integrin αvβ6, which is mainly regulated by the integrin β6 subunit gene (ITGB6), is a cell adhesion molecule that mediates cell‐cell and cell‐matrix interactions. However, the role of ITGB6 in the pathogenesis of IBD remains elusive. In this study, we found that ITGB6 was markedly upregulated in inflamed intestinal tissues from patients with IBD. Then, we generated an intestinal epithelial cell‐specific ITGB6 transgenic mouse model. Conditional ITGB6 transgene expression exacerbated experimental colitis in mouse models of acute and chronic dextran sulphate sodium (DSS)‐induced colitis. Survival analyses revealed that ITGB6 transgene expression correlated with poor prognosis in DSS‐induced colitis. Furthermore, our data indicated that ITGB6 transgene expression increased macrophages infiltration, pro‐inflammatory cytokines secretion, integrin ligands expression and Stat1 signalling pathway activation. Collectively, our findings revealed a previously unknown role of ITGB6 in IBD and highlighted the possibility of ITGB6 as a diagnostic marker and therapeutic target for IBD.

## 1 INTRODUCTION

Inflammatory bowel disease (IBD) refers to a group of chronic immune‐mediated disorders, encompassing ulcerative colitis (UC) and Crohn's disease (CD).^1^ It is characterized by chronic and recurring inflammation in the human gastrointestinal tract due to aberrant and inordinate inflammatory responses.^2^, ^3^ IBD patients often experience a lifelong course and display patterns of relapse and remission, with a variable degree of intensity from mild to severe. They may present with symptoms of diarrhoea, abdominal pain, weight loss or rectal bleeding.^1^, ^4^ It is a multifactorial disorder that is driven by the combination of genetic susceptibility, a disrupted epithelial barrier and a dysregulated immune response.^5^, ^6^, ^7^ Studies indicate that environmental risk factors include smoking, lack of physical activity, socioeconomic status, medication use, and pet exposure.^7^, ^8^ However, the cellular and molecular mechanisms contributing to IBD are still not completely understood. Therefore, further studies are required to elucidate the aetiology and pathogenesis of IBD.

Integrins refer to a large family of transmembrane α/β heterodimers, which function as cell anchoring and signal‐transducing receptors in a variety of physiological and pathological situations. Integrins are assembled by two noncovalently‐bound subunits, one α (1 of 18 α) subunit and one β subunit (1 of 8 β) in mammals. Hence, they can produce at least 24 integrin heterodimers, each of which can bind to a specific ligand.^9^, ^10^ They link the cell cytoskeleton to the extracellular matrix (ECM), and therefore mediate cell‐ECM and cell‐cell interactions.^10^ Integrins trigger multiple signalling pathways involving diverse biological processes, including adhesion, inflammation, migration, differentiation, senescence, cell division and cell metabolism.^11^, ^12^, ^13^, ^14^ Integrin αvβ6, whose expression is mainly controlled by the integrin subunit β6 gene (ITGB6), forms a heterodimer that consists of the αv and β6 subunits.^15^, ^16^ Studies provide evidence that integrin αvβ6 can recognize ligands that contain tripeptide arginine‐glycine‐aspartic acid sequence motifs, such as fibronectin, fibrinogen and vitronectin.^15^

In the present study, findings from clinical samples and public datasets revealed increased αvβ6 protein and *ITGB6* mRNA expression in human IBD tissues compared with that in adjacent normal tissues. We further generated conditional ITGB6 transgenic mice (Villin1‐ITGB6 Tg), and found that ITGB6 Tg mice exhibited increased susceptibility to acute and chronic DSS‐induced colitis. Conditional ITGB6 transgene expression also increased mortality in mice after DSS treatment. In addition, ITGB6 overexpression promoted the infiltration of macrophages, induced the secretion of pro‐inflammatory cytokines and integrin ligands, and activated the Stat1 signalling pathways. Together, these results revealed that ITGB6 might serve as a potential marker and promising therapeutic target for IBD.

## 2 MATERIALS AND METHODS

### 2.1 Human specimens

All clinical specimens used in this study were obtained from the Second Affiliated Hospital of Zhejiang University School of Medicine between September 2016 and July 2017 from 17 patients who were diagnosed with IBD and underwent surgical resection. We collected the inflamed and uninflamed small or large intestinal tissues for each patient from the surgical resection specimens. Informed consent was obtained from all the patients. The protocol was approved by the Ethical Committee of the Second Affiliated Hospital of Zhejiang University School of Medicine.

### 2.2 GEO data analysis

The expression data of GSE11223, GSE20881 and GSE38713 were downloaded from the Gene Expression Omnibus (GEO) (http://www.ncbi.nlm.nih.gov/geo/). They were used to compare the expression level of ITGB6 between inflamed and uninflamed tissues, and to investigate the association between the ITGB6 expression and clinical manifestations of patients.

### 2.3 Generation of Villin1‐ITGB6 transgenic mice

To generate IEC‐specific ITGB6 Tg mice, we constructed a Villin1‐ITGB6 plasmid that included the coding region of full‐length *ITGB6* cDNA under the control of the *Villin1* promoter. Specifically, the plasmid construct contained the *Villin1* promoter, the full‐length coding sequence of *ITGB6*, a linker and the green fluorescent protein coding region (*ZsGreen*). Then, the plasmid was introduced into the C57BL/6 mouse genome by pronuclear microinjection, and Tg founder mice were generated. Then the founder lines were crossed with the wild‐type C57BL/6 inbred line (purchased from the Model Animal Research Center of Nanjing University). For genotyping, genomic DNA was isolated from mouse tails, and a polymerase chain reaction (PCR) assay was carried out using the four primers listed in Table S1. The mice were housed in a specific pathogen‐free facility under a 12‐hour light and 12‐hour dark cycle in the Zhejiang University Laboratory Animal Center. The Animal Care and Use Committee of the Zhejiang University School of Medicine approved all experimental procedures involving animals.

### 2.4 Mouse model of DSS‐induced colitis

Acute DSS‐induced colitis was induced in Tg and C57BL/6 WT mice by administering 2.5% (wt/vol) DSS (MP Biomedicals) dissolved in drinking water to age‐ and sex‐matched mice for 7 days. Body weight, stool consistency and rectal bleeding were recorded once per day. These factors constituted the disease activity index (DAI) (Table S2). Specifically, the DAI score was calculated by combinations of scores of weight loss, stool consistency and gross bleeding (range 0‐12). On day 7, the entire colon was removed and colon length from the caecum to the anus was measured. For histological analysis, colons were isolated, fixed in formalin solution, embedded in paraffin, and cut into 4‐μm sections. Then they were stained with haematoxylin‐eosin (H&E) solutions (Thermo Fisher Scientific) or a periodic acid‐Schiff (PAS) staining kit (IMEB). For survival analysis, Tg and WT mice were provided with 2.5% or 3% DSS for the indicated number of days.

For chronic DSS‐induced colitis, mice were orally exposed to four‐cycle DSS treatment. During each cycle, 1.5% DSS was dissolved in drinking water for 4 days, followed by a 4‐day interval of normal drinking water. Body weight and gross bleeding were recorded every other day, and colon length was measured at the end of the experiment.

### 2.5 Isolation of intestinal epithelial cells or lamina propria cells

Intestines were dissected, washed in PBS supplemented with antibiotics and cut into 2‐mm pieces. Then, tissues were incubated in extraction buffer (10 mL of HBSS containing 5 mM EDTA and 1 mM DTT) at 37°C for 30 minutes. For isolation of IECs, the tubes were shaken forcefully to release epithelial cells, which were repeated three times, followed by centrifugation at 1000 *g* at 4°C for 10 minutes. After removing epithelial cells, the remaining fragments were digested in HBSS (Gibco) supplemented with 0.5% collagenase D (Sigma) and 0.05% DNase (Sigma) at 37°C for 30 minutes. The supernatants were filtered through 70‐μm cell strainers to collect lamina propria cells.

### 2.6 RNA extraction, reverse transcription and quantitative real‐time PCR (qPCR)

Total RNA was isolated by TRIzol reagent (Invitrogen) according to the manufacturer's standard protocol. Following quantification, total RNA was reverse‐transcribed to cDNA with a PrimeScript RT reagent kit (Takara, CHN). qPCRwas performed using Thunderbird SYBR Master Mix (Takara, CHN) in accordance with the manufacturer's guidelines. The primers are summarized in Table S3. GAPDH was used as an internal control. For quantification, the $2^{‐\Delta \Delta C_{t}}$ method was used and Student's *t*‐test was used for statistical analysis.

### 2.7 Cytokine array

RT^2^ Profiler™ PCR Array Mouse Inflammatory Response & Autoimmunity (PAMM‐077Z) was used to detect relative expression of cytokines from intestinal tissue between WT and Tg mice after DSS treatment. RT^2^ Profiler™ PCR Array profiled the expression of 84 key genes involved in autoimmune and inflammatory immune responses. In brief, total RNA was extracted from intestinal tissue, reversely transcribed into cDNA and further subjected to qPCR assay. The $2^{‐\Delta \Delta C_{t}}$ method was used for quantification and Student's *t*‐test was used for statistical analysis.

### 2.8 Flow cytometry

The expression of surface markers was analysed by flow cytometry. Cells were harvested and washed. Then they were stained with APC‐conjugated anti‐CD45 (Thermo Fisher Scientific), eFluor 450‐conjugated anti‐CD3 (Thermo Fisher Scientific) or PE‐conjugated anti‐CD68 (Thermo Fisher Scientific) in the dark on ice for 1 hour. The cells were then centrifuged at 650 *g* for 3 minutes and the supernatant was discarded. Following resuspension, single cells were assayed by a flow cytometer (Beckman Coulter) and analysed using FlowJo V10 software (Treestar). The experiments were repeated at least three times.

### 2.9 Statistical analysis

The data are present as the means with error bars depicting the standard deviations or standard errors of the mean. Student's *t*‐test and one‐way ANOVA were used to determine the significant differences. Survival curves were generated by Kaplan‐Meier analysis. *P* < 0.05 was considered significant. All statistical analyses were carried out using SPSS 20.0 software (SPSS Inc) or GraphPad Prism 7.0 software.

## 3 RESULTS

### 3.1 ITGB6 expression was elevated in human IBD tissues and positively correlated with disease activity

**FIGURE 1:**
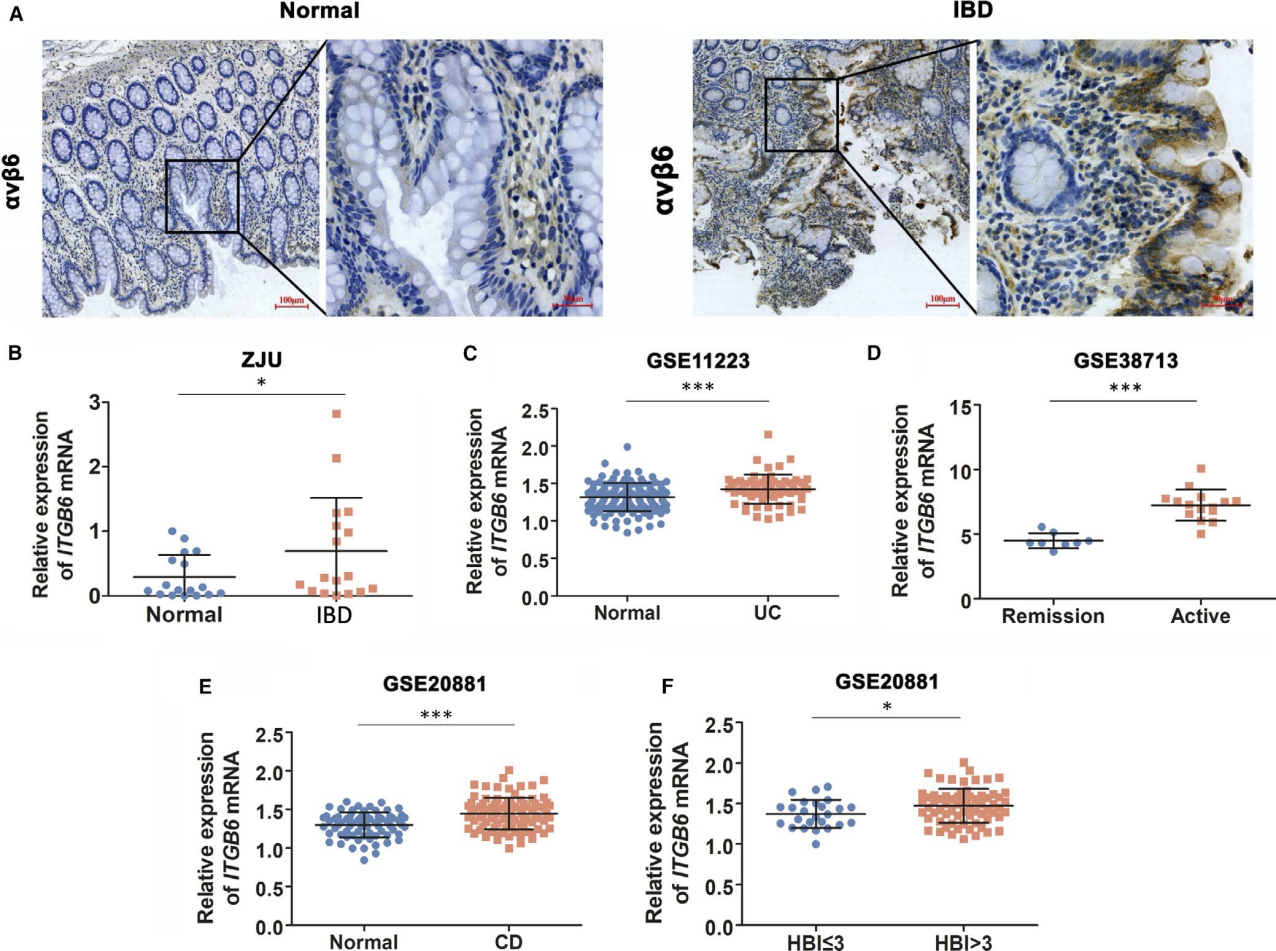
ITGB6 was upregulated in human IBD tissues and positively correlated with disease activity. A, Representative IHC images of αvβ6 protein expression in inflamed tissues and matched adjacent normal tissues in large intestines from patients with IBD in ZJU center. Scale bar, 100 μm or 30 μm. B, qPCRanalysis of *ITGB6* mRNA levels in 17 pairs of human IBD tissues and matched adjacent normal small or large intestinal tissues from ZJU center, and they were compared regardless of their origin. Data are presented as the mean ± SD. C, Expression levels of *ITGB6* mRNA in patients with UC vs those in normal controls from the GSE11223 dataset. D, Expression levels of *ITGB6* mRNA in patients with UC in the remission phase vs those in patients with UC in the active phase from the GSE38713 dataset. E‐F, Expression levels of *ITGB6* mRNA (E) in patients with CD vs those in normal controls and; (F) in patients with CD with Harvey Bradshaw index (HBI) ≤ 3 vs those in patients with CD with HBI > 3 from the GSE20881 dataset. Data are presented as the mean ± SD. *P < 0.05, ***P < 0.001

To investigate the potential significance of αvβ6 in IBD, we detected the expression of αvβ6 in inflamed and adjacent uninflamed intestines in IBD patients in our center (ZJU). As shown in Figure 1A, αvβ6 was rarely expressed in the large intestine, but overexpressed in IECs of IBD patients. Further qPCR analysis showed that mRNA level of *ITGB6* was significantly higher in inflamed small and large intestines in patients with IBD (*P* < 0.05, Figure 1B and Table S4). Considering that most of the included patients in our center had CD, we further explored the association of *ITGB6* expression and IBD by data mining of public gene expression data. Consistently, the mRNA levels of *ITGB6* showed a marked increase in not only CD patients but also UC patients compared with those in controls from GSE11223 datasets (*P* < 0.001, Figure 1C and Table S5‐1). Furthermore, *ITGB6* was dramatically upregulated in intestinal tissues from UC patients in the active phase compared with that in intestinal tissues from UC patients in remission (GSE38713, *P* < 0.001, Figure 1D and Table S5‐2). The Harvey Bradshaw index (HBI) is a simplified index to assess the disease activity of CD,^17^ including five parameters: general well‐being, abdominal pain, daily number of liquid stools, abdominal mass and complications.^18^ Consistent with the observation in UC, substantially higher levels of *ITGB6* were found in CD patients (*P* < 0.001, Figure 1E and Table S5‐1) than normal controls, and in CD patients with HBI > 3 than in patients with HBI ≤ 3 from the GSE20881 dataset (*P* < 0.05, Figure 1F). In summary, ITGB6 was elevated in small and large intestines from patients with IBD and was positively correlated with the severity of IBD, implying that ITGB6 might play an essential role in the regulation of inflammation and tissue damage in IBD.

### 3.2 Generation and characterization of the ITGB6 conditional transgenic mouse model

**FIGURE 2:**
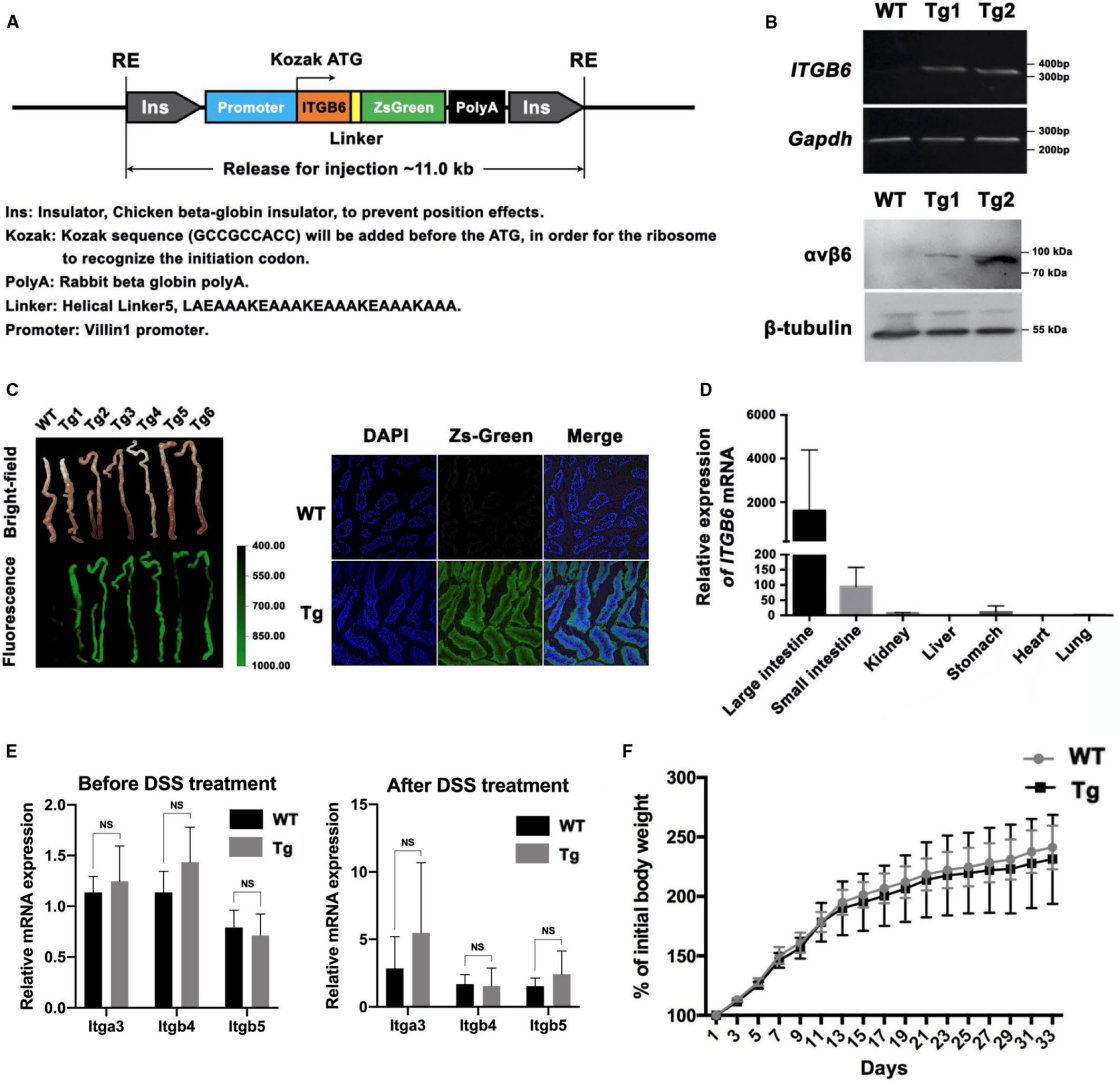
Generation and characterization of the ITGB6 conditional transgenic mouse model. A, IEC‐specific transgenic mouse construction strategy and schematic diagram of the ITGB6 transgene construct under the control of the Villin1 promoter. B, PCR analysis of *ITGB6* mRNA and WB analysis of αvβ6 expression in intestinal tissues from WT and two lines of Tg mice. C, Immunofluorescence analysis of Zs‐Green staining in the intestine tissues from WT and Tg mice. D, qPCRanalysis of *ITGB6* expression in the large intestine, small intestine, kidney, liver, stomach, heart and lung tissues from Tg mice (N = 5). E, The results of qPCR showed that the overexpression of ITGB6 in IECs didn't affect the expression of other integrins before or after DSS treatment (N = 5). F, The body weight change was not significantly different between WT mice and Tg mice (N = 5). NS, no significance

To explore the role of ITGB6 in IBD progression, we generated Tg mice conditionally overexpressing human ITGB6 in IECs under the control of the IEC‐specific *Villin‐1* promoter (Villin1‐ITGB6 Tg). A schematic diagram of the ITGB6 transgene is shown in Figure 2A *ITGB6* mRNA and αvβ6 protein expression levels were increased in the intestines of Tg mice compared to those in the intestines of WT mice (Figure 2B). Notably, immunofluorescence assays confirmed the strong expression of ZsGreen in Tg mice (Figure 2C). qPCR analysis also demonstrated that substantially higher *ITGB6* expression was found in the intestinal tissues, whereas low or nearly absent levels of *ITGB6* were observed in the kidney, liver, stomach, heart and lung tissues (Figure 2D).

Other epithelial integrins were also assessed by qPCR. As shown in Figure 2E, the mRNA levels of *Itga3, Itgb4* and *Itgb5* in the intestine were not significantly different between WT mice and Tg mice treated with or without DSS. Body weights and faecal occult blood (FOB) scores of mice were also monitored for four weeks (beginning at 3 weeks of age) to investigate any possible changes in growth and development as well as stool after transgenic *ITGB6* expression. There was no significant difference in body weight (Figure 2F) or FOB score (data not shown) between WT mice and Tg mice. Further HE examination did not reveal detectable histopathological changes in the large intestines of Tg mice (Figure S1A). In addition, the infiltration of CD45^+^ immune cells (Figure S1B) and of the subpopulations of CD3^+^ T cells (Figure S1C) and CD68^+^ macrophages (Figure S1D) was identical between Tg mice and the control WT mice. Overall, we successfully generated a Tg mouse displaying conditional overexpression of ITGB6 in IECs and showing no histopathological features.

### 3.3 ITGB6 Tg mice displayed increased susceptibility to acute DSS‐induced colitis

**FIGURE 3:**
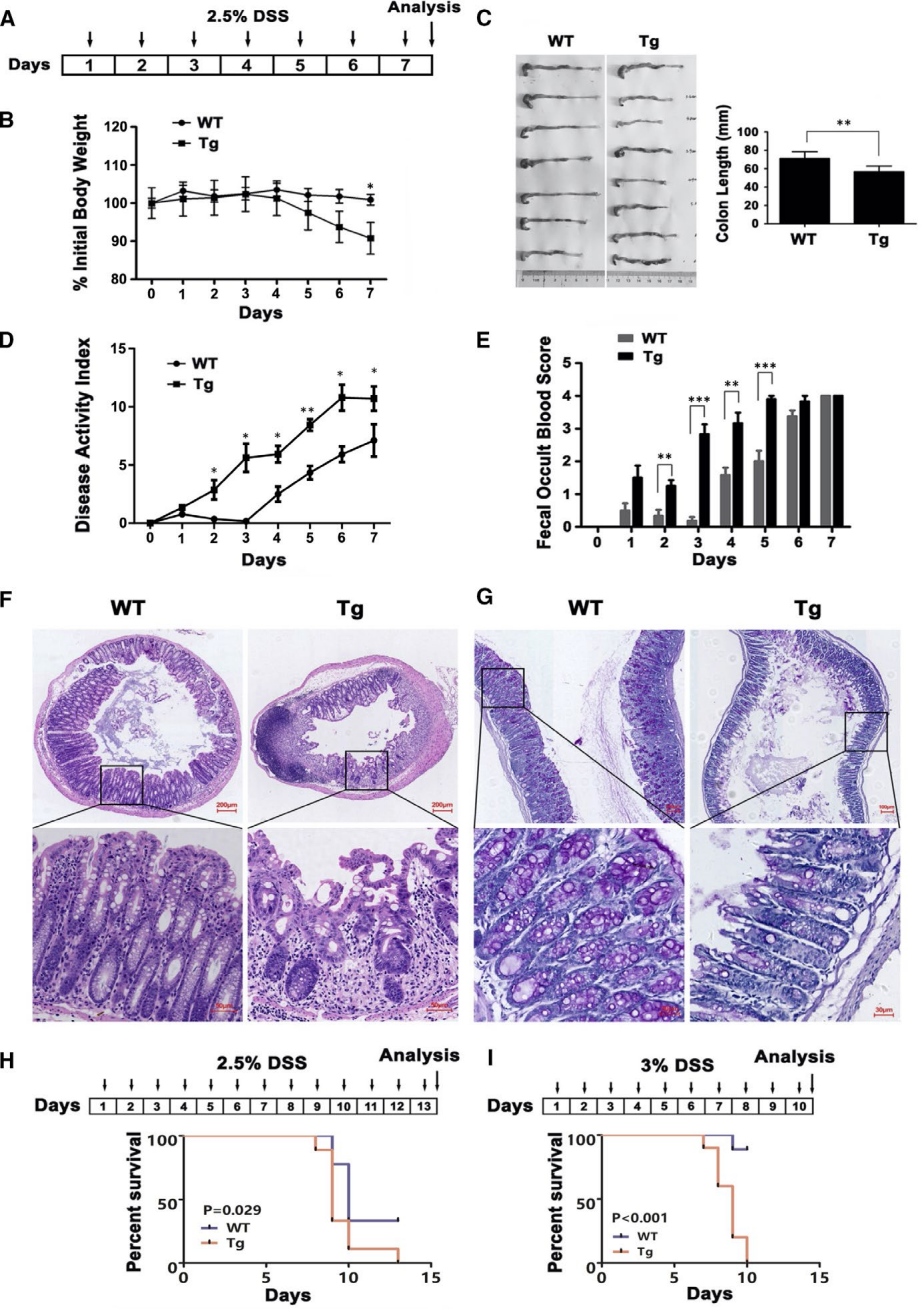
ITGB6 Tg mice exhibited more susceptibility to acute DSS‐induced colitis. A, Experimental schedule of acute DSS‐induced colitis. WT and Tg mice were provided 2.5% DSS in the drinking water for 7 d. Mice were sacrificed on day 7. B‐E, (B) Body weight changes (% original body weight, mean ± SEM); (C) representative colon images (left) and quantitative colon length measurements (right, mean ± SD); (D) disease activity index (mean ± SD); and (E) faecal occult blood scores (mean ± SEM) are shown. Values were compared between the WT and Tg groups. F, Representative images of H&E staining of colonic sections in WT and Tg mice after DSS treatment on day 7. Scale bar, 200 μm or 50 μm. G, Representative images of PAS staining of colonic sections in WT and Tg mice after DSS treatment on day 7. Scale bar, 100 μm or 30 μm. H, Kaplan‐Meier survival plots of WT (N = 9) and Tg mice (N = 9) following 2.5% DSS treatment for 13 d. The scheme of the experimental design for DSS administration is presented in the top panel. I, Kaplan‐Meier survival plots of WT (N = 9) and Tg (N = 10) mice following 3% DSS treatment for 10 d. For all the above results, three independent experiments were performed. *P < 0.05, **P < 0.01, ***P < 0.001

To investigate the role of ITGB6 in the development of IBD, we used the DSS model, which chemically induced UC‐like inflammation. As illustrated in Figure 3A, 2.5% DSS solution was administered to mice in their drinking water for 7 days. The body weights of ITGB6 Tg mice were significantly lower than those of WT mice on day 7 (*P* < 0.05), with a similar trend occurring during the first few days (Figure 3B). With respect to colon length, ITGB6 Tg mice displayed decreased colon length compared with that of control mice on day 7 (*P* < 0.01, Figure 3C). DAI scores, based on a scoring system used to evaluate the severity of colitis, were increased by the administration of DSS in both WT and Tg mice during the course of the experiment. However, starting at day 2 post‐treatment, the DAI was significantly higher in Tg mice than in WT mice (Figure 3D). Similarly, FOB scores were also significantly increased in ITGB6 Tg mice compared to those in WT mice on days 2‐5 (Figure 3E).

Furthermore, we performed H&E and PAS staining of the colon sections to investigate the pathological changes in the DSS model. H&E staining indicated that ITGB6 Tg mice displayed more severe mucosal erosions, ulcers, and crypt destruction in the colon sections than mice (Figure 3F). Consistently, the findings from PAS staining showed much more depletion of mucous‐producing goblet cells in the colon tissues from ITGB6 Tg mice (Figure 3G).

To investigate the effect of ITGB6 on survival in acute colitis, WT and Tg mice were administrated 2.5% DSS in drinking water for 13 days or 3% DSS for 10 days and then survival rates were determined. Based on Kaplan‐Meier survival analysis, our results indicated that Tg mice exhibited increased mortality in comparison with that of WT mice when treated with 2.5% DSS (*P* < 0.05, Figure 3H) or 3% DSS‐induced colitis (*P* < 0.001, Figure 3I). Overall, these results suggested that ITGB6 influenced susceptibility to acute colitis in mice and exacerbated the severity of acute DSS‐induced colitis.

### 3.4 ITGB6 Tg mice exhibited increased susceptibility to chronic DSS‐induced colitis

**FIGURE 4:**
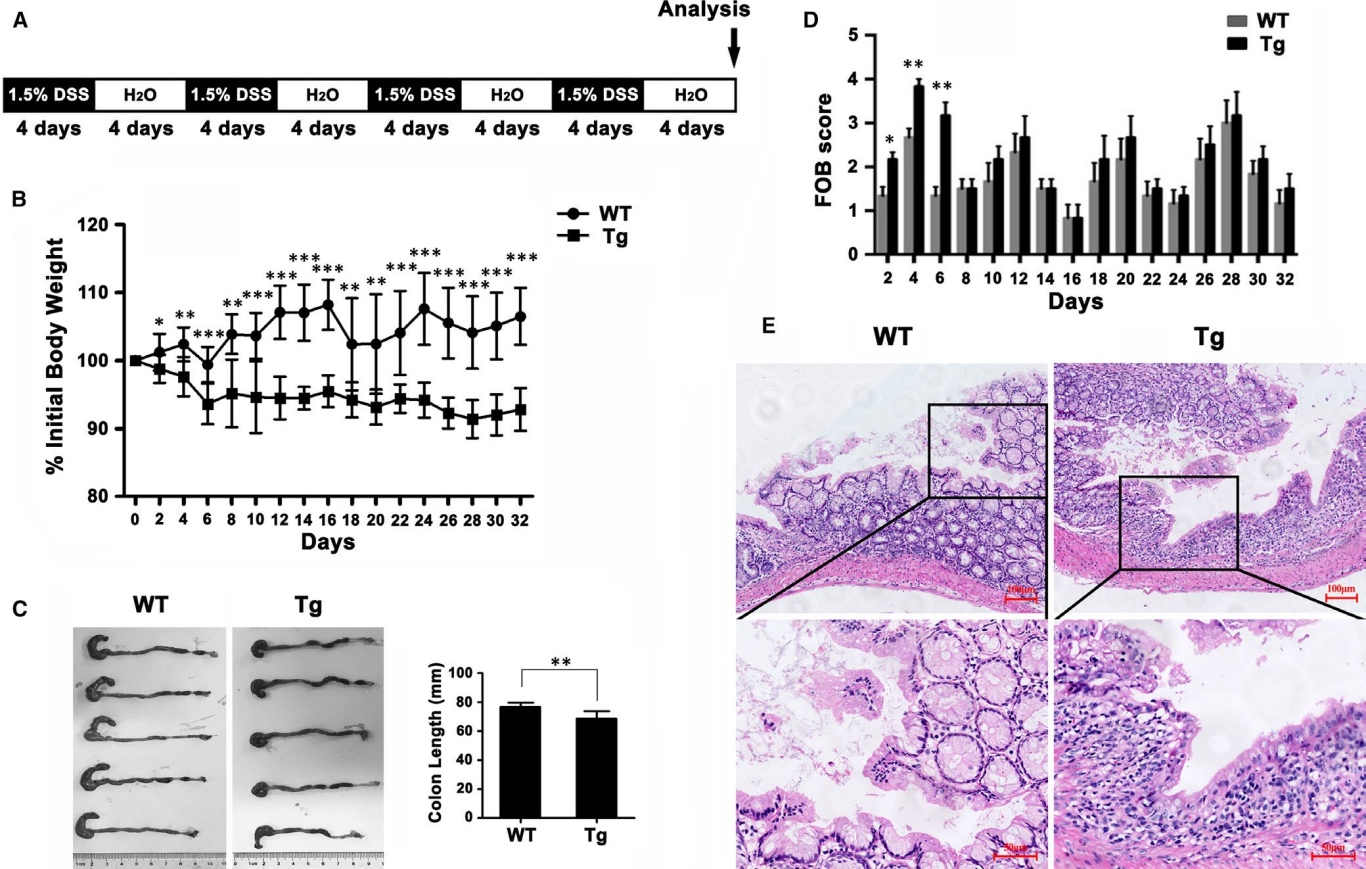
ITGB6 Tg mice exhibited increased susceptibility to chronic DSS‐induced colitis. A, Experimental schedule of chronic DSS‐induced colitis. WT and Tg mice were provided four cycles of 1.5% DSS for 4 d and then normal drinking water for another 4 d. B‐D, (B) Body weight changes (% original body weight, mean ± SD); (C) representative colon images (left) and quantitative colon length measurements (right, mean ± SD); and (D) faecal occult blood score are shown (mean ± SEM). Values were compared between the WT and Tg groups. E, Representative images of H&E staining of colonic sections in WT and Tg mice after DSS treatment on day 32. Scale bar, 100 μm or 50 μm. For all the above results, three independent experiments were performed. *P < 0.05, **P < 0.01, ***P < 0.001

To further delineate the contribution of conditional overexpression of ITGB6 in IECs to chronic DSS‐induced colitis, WT and Tg mice were provided 1.5% DSS from days 1 to 4 and then normal water intake from days 5 to 8 for four cycles (Figure 4A). The pathological changes reflecting colitis between the Tg and the control groups were analysed. In accordance with the differences observed in the acute DSS‐induced colitis model, Tg mice presented more obvious body‐weight loss (Figure 4B), shorter colon length (Figure 4C), and more stool bleeding than WT mice (Figure 4D). Histologically, lesions in the mucosal and submucosal areas of the colon were more severe in Tg mice (Figure 4E). Taken together, these results demonstrated that ITGB6 transgene expression aggravated chronic DSS‐induced colitis in mice.

### 3.5 ITGB6 promoted macrophage infiltration in the model of DSS‐induced colitis

**FIGURE 5:**
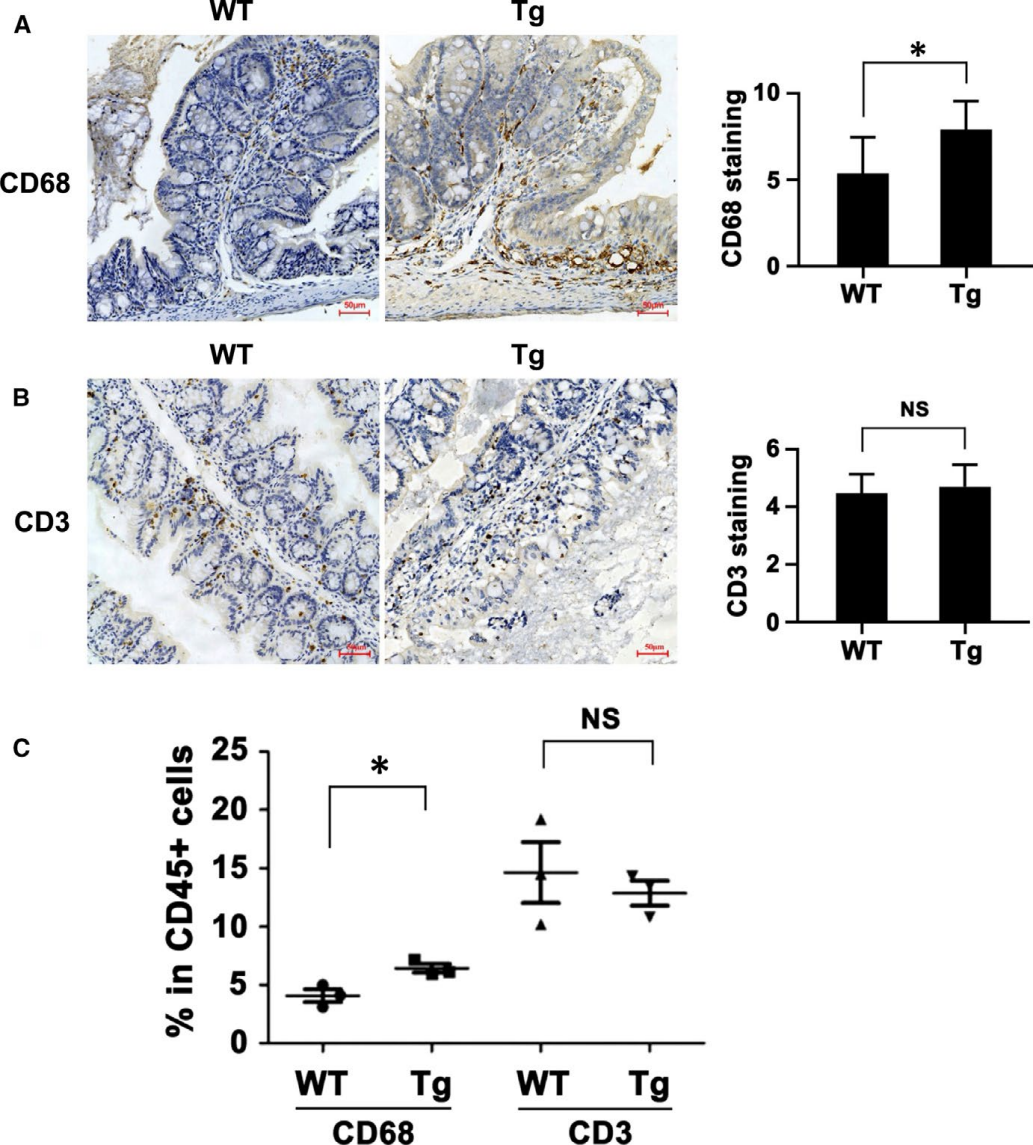
Conditional overexpression of ITGB6 in IECs promoted the infiltration of macrophages. A‐B, Representative images (left) and quantification (right) of IHC staining of (A) CD68 and (B) CD3 in colons from WT and Tg mice. Scale bars, 50 μm. Data are presented as the mean ± SD. C, Flow cytometric analysis of the percentage of macrophage (% CD68^+^ in CD45^+^ cells) and T cells (% CD3^+^ in CD45^+^ cells) in the lamina propria of the colons from WT and Tg mice. Data are presented as the mean ± SEM. *P < 0.05. NS, no significance

As reported, IBD was associated with the disturbance of immune cell homeostasis. The previous results indicated that ITGB6 promoted the progression of IBD. Therefore, in this part, we investigated the role of ITGB6 in the recruitment of inflammatory cells in a DSS‐induced colitis model. First, we performed IHC analysis to evaluate the infiltration of macrophages (CD68^+^) and T cells (CD3^+^) in colon tissues from WT and Tg mice. Tg mice showed a pronounced accumulation of macrophages when compared to that of controls mice (Figure 5A, *P* < 0.05). However, IHC analysis of CD3 staining demonstrated that ITGB6 did not significantly influence T‐cell accumulation (Figure 5B). Then, we performed flow cytometry to investigate the percentage of macrophages (CD45^+^CD68^+^) and T cells (CD45^+^CD3^+^) in the lamina propria of mice after DSS treatment. As shown in Figure 5C, an elevated infiltration of CD45^+^CD68^+^ macrophages was observed in ITGB6 Tg mice compared with that in WT mice (*P* < 0.05). However, the percentage of T cells exhibited no appreciable difference between the two groups (Figure 5C). These results suggested that ITGB6 facilitated macrophage infiltration, which might explain the exacerbated intestinal inflammation.

### 3.6 ITGB6 dysregulated the secretion of pro‐inflammatory cytokines and integrin ligands in the model of DSS‐induced colitis

**FIGURE 6:**
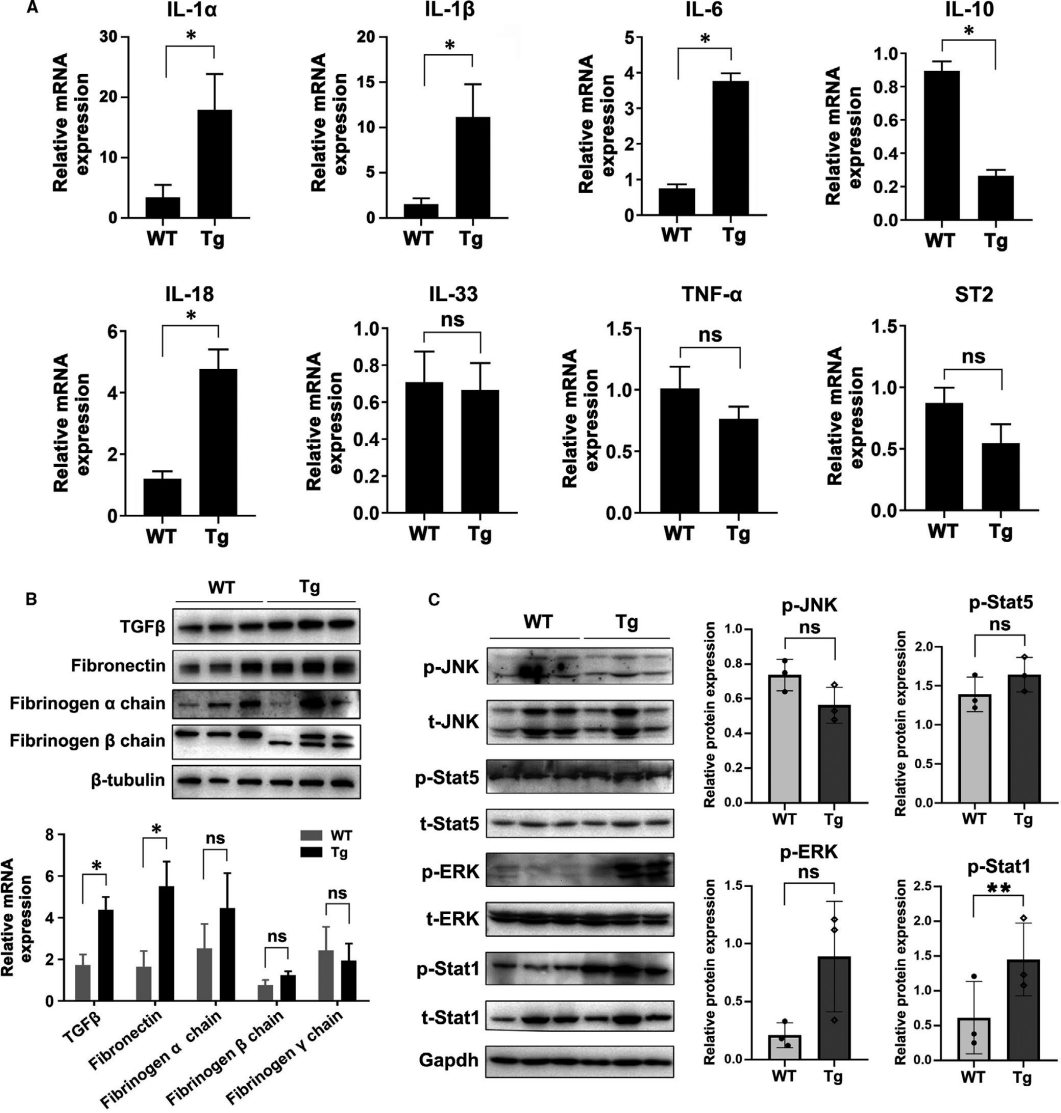
ITGB6 dysregulated the secretion of pro‐inflammatory cytokines and integrin ligands in the model of DSS‐induced colitis. A, qPCR analysis of *IL‐1α*, *IL‐1β, IL‐6, IL‐10, IL‐18, IL‐33, TNF‐a* and *ST2* mRNA levels in colons from WT and Tg mice. Data are presented as the mean ± SEM. B, qPCR and WB analysis of TGF‐β, fibronectin and fibrinogen in colons from WT and Tg mice. C, WB analysis of the phosphorylated and total Stat1, Stat5, JNK, and ERK in IECs from WT and Tg mice. WB quantifications of phosphorylated proteins were shown on the right. Gapdh was used as an internal control. *P < 0.05, **P < 0.01, NS, no significance

The altered infiltration of macrophages will result in the release of a variety of pro‐inflammatory cytokines, and the complex and dynamic cytokine network plays an important role in regulating mucosal innate and adaptive immune responses in IBD. To explore the cytokine alterations, we performed a cytokine array by using colon tissues from Tg and WT mice after DSS treatment. Of note, a few cytokines were dysregulated in Tg mice when compared to WT mice (Table S6). We validated some of the cytokines known to be involved in IBD pathogenesis by qPCR. Specifically, we found the increased levels of *IL‐1α* (*P* < 0.05), *IL‐1β* (*P* < 0.05), *IL‐6* (*P* < 0.05) and *IL‐18* (*P* < 0.05). Meanwhile, *IL‐10* was significantly decreased (*P* < 0.05), and no significant difference was observed for *IL‐33, St2* and *TNF‐a* (Figure 6A). ITGB6 binds to and activates the latent form of TGF‐β, and can mediate cell‐ECM interactions. Besides, integrin ligands also participate in pathogenesis of IBD. As ligands of ITGB6, TGF‐β and fibronectin were significantly increased in the colon of DSS‐treated Tg mice (Figure 6B). Although Kindlin‐1 promotes ITGB6‐mediated TGF‐β activation and release, we didn't identify the significant expression difference of Kindlin‐1 between the colon of Tg mice and WT mice in DSS‐induced colitis model (data not shown).

Additionally, the Stat1, Stat5, JNK and ERK signalling pathways contribute to pro‐inflammatory effects. Thus, we investigated the phosphorylation levels of these proteins, including p‐Stat1, p‐Stat5, p‐JNK and p‐ERK, in IECs from Tg and WT mice after DSS treatment. As illustrated in Figure 6C, ITGB6 transgene expression significantly promoted the phosphorylation of Stat1 in IECs. However, no significant difference in p‐JNK, p‐ERK, p‐Stat5 levels was observed between the two groups (Figure 6C). Collectively, these data suggested that the ITGB6 transgene dysregulated the secretion of pro‐inflammatory cytokines, upregulated the expression of integrin ligands and the phosphorylation of Stat1 in DSS‐induced colitis in mice, which might contribute to DSS‐induced colitis.

## 4 DISCUSSION

Inflammatory bowel diseases are lifelong immunologically mediated disorders of the digestive tract, and are increasingly prevalent worldwide.^1^, ^7^ Although there is emerging knowledge about its molecular mechanisms, IBD remains one of the most fascinating and challenging diseases worldwide. In this study, we focused on the identification of key regulators involved in IBD.

In the normal state, the gastrointestinal tract maintains homeostasis by regulating immune responses to defend against foreign microbial antigens and a diverse array of antigens in food. In contrast, the disruption of this homeostasis in the gastrointestinal tract is believed to cause IBD.^19^ Our study showed that ITGB6 was overexpressed in IECs of IBD patients and correlated with disease activity. IECs are some of the most crucial components of the intestinal barrier, functioning in not only the physical and immunological barriers, but also nutrient absorption. Dysregulation of IECs contributes to the outbalanced immune responses toward luminal antigens, and the development of IBD. Therefore, in this study, we generated a Tg mouse conditionally overexpressing human ITGB6 in IECs, and a validated DSS‐induced colitis model mimicking the complex UC seen in patients to explore the role of ITGB6 in IBD progression.

Unsurprisingly, we found that ITGB6 Tg mice exhibited increased susceptibility to acute and chronic DSS‐induced colitis. Integrins represent a large family of transmembrane glycoproteins that bind to laminin receptors, leukocyte receptors, collagen receptors and arginine‐glycine‐aspartic acid receptors to propagate signals.^20^ In collaboration with various types of receptors, integrins have the ability to maintain tissue homeostasis and modulate multiple biological processes, such as proliferation, survival and migration.^14^ De Arcangelis et al^21^ reported that IEC‐specific ablation of α6β4 integrin results in predisposition to IBD. Besides, α4β7 integrin is believed to be closely linked to the aetiology of IBD.^22^ During an inflammatory response, they help direct leukocyte trafficking into sites of inflammation in the gastrointestinal tract.^23^, ^24^ This homing process is mediated by α4β7 integrin, which has been identified as a receptor for mucosal addressin cellular adhesion molecule‐1 (MAdCAM‐1), a known endothelial cell adhesion molecule on the intestinal vascular endothelium.^25^ Intriguingly, several anti‐integrin therapies have proven to be effective at inhibiting the extravasation of leukocytes in IBD, including antibodies against α4 (eg, natalizumab and AJM300), antibodies against α4β7 specifically (eg, vedolizumab and abrilumab) and antibody against β7 (eg, etrolizumab).^26^, ^27^, ^28^, ^29^, ^30^

Unlike α4β7, which is highly expressed in circulating lymphocytes, αvβ6 is an epithelium‐restricted cell surface receptor and is highly expressed in IECs. It was also reported to be dramatically upregulated in response to inflammatory stimuli.^15^, ^16^, ^31^, ^32^ Generally, IBD is considered to exhibit the remarkable property of hyperactivation of inflammatory processes, characterized by the production of pro‐inflammatory cytokines and chemokines, and recruitment and activation of intestinal immune cells.^33^ Increasing evidence supports that the IL‐1 family plays vital roles in IBD due to its pro‐inflammatory capacities. McAlindon et al reported that IBD colonic macrophages promoted the activation of the IL‐1β‐converting enzyme and the release of mature IL‐1β, thereby inducing pro‐inflammatory effects and playing crucial roles in IBD.^34^ Moreover, in their study, by assessing the role of IL‐1β in two mouse models of intestinal inflammation, Coccia et al found that IL‐1β could promote immune responses by facilitating the recruitment of granulocytes and accumulation of innate lymphoid cells (ILCs).^35^ IL‐18, as a member of the IL‐1 family of cytokines, also contributed to IBD development. It was shown that an IL‐18 transgene produced increased susceptibility to DSS‐induced colitis.^36^ Consistently, blockade of IL‐18 by administration of a monoclonal antibody or anti‐sense *IL‐18* mRNA could alleviate DSS‐ and trinitrobenzene sulfonic acid‐induced colitis in mice due to decreased production of IFN‐γ and TNF.^37^ IL‐6 was also identified to be positively correlated with severity of colitis in several mouse models.^38^ On the contrary, IL‐10 is an crucial mediator of the regulatory functions of T cells and has a significant anti‐inflammatory effect in the gastrointestinal tract.^39^ Here, we found that the levels of *IL‐1α*, *IL‐1β*, *IL‐18* and *IL‐6* were significantly higher in Tg group than in the control group, while the level of *IL‐10* was significantly lower, which was consistent with other studies. However, we did not find any significant change of *TNF‐a,* and *ST2/IL‐33* axis in our study, which is also reported to regulate the inflammatory process in IBD.^40^ Although cytokines produced by immune cells have been implicated to amplify and sustain immune response in intestines,^41^, ^42^ the cytokine network is complex and dynamic.^41^, ^42^ The altered cytokines are not mutually exclusive and it's difficult to identify the exact one that contributes to disease. Besides, it's sometimes not unambiguous and the individual cytokine can have opposing functions in different clinical and immunological settings.^43^

As the critical intercellular messengers of the immune response, cytokines regulate immune cell homeostasis. Apart from the increased pro‐inflammatory cytokines in Tg mice, we found that ITGB6 facilitated macrophage infiltration to exacerbate intestinal inflammation in the mouse model. Mounting evidence suggests that various types of immune cells, such as macrophages, dendritic cells, Treg cells, T helper cells and others, mediate intestinal homeostasis and inflammation.^19^ As the first line of defence of the immune system against pathogens, intestinal macrophages located in the subepithelial lamina propria clear pathogens to exert the functions of innate immunity, and they also serve as antigen‐presenting cells for crosstalk with T cells to exert the functions of adaptive immunity.^44^ Of interest, two subsets of macrophages, known as resident macrophages and inflammatory macrophages, perform functions to maintain intestinal homeostasis.^45^, ^46^ Accumulating evidence from several mouse models of colitis suggests the contribution of Ly6C^hi^ monocytes to the development of IBD.^47^, ^48^ These cells overproduce pro‐inflammatory cytokines and hence lead to dysregulated inflammatory responses.^45^, ^49^, ^50^

TGF‐β is the ligand of ITGB6 and it is activated by combining with ITGB6, while the maintenance of ITGB6 expression also requires the existence of TGF‐β. The ITGB6‐TGF‐β interplay forms a mutual positive feedback loop.^15^ Dysregulated TGF‐β signalling is associated with the development of intestinal inflammation in mouse models and IBD patients. In addition, Kindlin‐1 is reported to be an intracellular activator of ITGB6, and triggers ITGB6‐mediated TGF‐β activation and release.^15^ In this study, significant increase of TGF‐β was observed in large intestinal tissues, but we did not find the significant change of Kindlin‐1, which implies that it might be a kindlin‐1 independent increase of TGF‐β activated by ITGB6 in DSS‐induced colitis model. In addition to TGF‐β, fibronectin, which is the ECM ligands of ITGB6, was also significantly elevated in DSS‐treated Tg mice. Some previous studies have shown that ECM synthesis was increased and ECM remodelling was correlated with intestinal inflammation in IBD.^51^ For example, fibronectin, one of the components of submucosal ECM molecules, is dramatically increased in IBD and is associated with the altered cellular compositions of the submucosa in IBD.^51^ Besides, we also found that ITGB6 activates Stat1 signalling pathway. However, the exact molecular mechanisms by which ITGB6 exacerbates DSS‐induced colitis require further exploration. In conclusion, in this study, our results indicated that ITGB6 was significantly upregulated in inflamed IECs in IBD patients, and positively correlated with disease activity. Then, the conditional transgene of ITGB6 in IECs promoted IBD progression and increased the infiltration of macrophages, the secretion of pro‐inflammatory cytokines, the upregulation of integrin ligands and the activation of Stat1 signalling pathway. Future studies are warranted to investigate the underlying mechanisms by which ITGB6 promotes the immune response in DSS‐induced colitis in mice. Such studies will provide additional insights into IBD and provide a rationale for the utilization of innovative therapy in targeting ITGB6 to improve IBD treatment.

## CONFLICT OF INTEREST

All the authors declare no conflict of interest.

## AUTHOR CONTRIBUTIONS

**Haiyan Chen:** Data curation (equal); Formal analysis (equal); Writing‐review & editing (equal). **Liubo Chen:** Data curation (equal); Formal analysis (equal); Writing‐review & editing (equal). **Xin Wang:** Methodology (equal); Resources (equal); Writing‐review & editing (equal). **Xiaoxu Ge:** Methodology (equal); Resources (equal); Writing‐review & editing (equal). **Lifeng Sun:** Methodology (equal); Resources (equal); Writing‐review & editing (equal). **Zhanhuai Wang:** Methodology (equal); Resources (equal); Writing‐review & editing (equal). **Xiaoming Xu:** Resources (equal); Writing‐review & editing (equal). **Yongmao Song:** Methodology (equal); Resources (equal); Writing‐review & editing (equal). **Jing Chen:** Methodology (equal); Resources (equal); Writing‐review & editing (equal). **Qun Deng:** Methodology (equal); Resources (equal); Writing‐review & editing (equal). **Haiting Xie:** Methodology (equal); Resources (equal); Writing‐review & editing (equal). **Ting Chen:** Funding acquisition (equal); Methodology (equal); Resources (equal); Writing‐review & editing (equal). **Yan Chen:** Methodology (equal); Resources (equal); Writing‐review & editing (equal). **Kefeng Ding:** Conceptualization (equal); Project administration (equal); Supervision (equal); Writing‐original draft (equal); Writing‐review & editing (equal). **Jingjing Wu:** Conceptualization (equal); Funding acquisition (equal); Project administration (equal); Supervision (equal); Writing‐original draft (equal); Writing‐review & editing (equal). **Jian Wang:** Data curation (equal); Formal analysis (equal); Funding acquisition (equal); Methodology (equal); Writing‐review & editing (equal).

## Supporting information

Fig S1

Supplementary Material

## Data Availability

The datasets used and/or analysed during the current study are available from the corresponding author upon reasonable request.
