# Supplementary figures and images for: Transgenic overexpression of ITGB6 in intestinal epithelial cells exacerbates dextran sulfate sodium‐induced colitis in mice
Source: J Cell Mol Med. 2021 Jan 24;25(5):2679–90. doi: 10.1111/jcmm.16297 (PMC7933932; doi:10.1111/jcmm.16297)

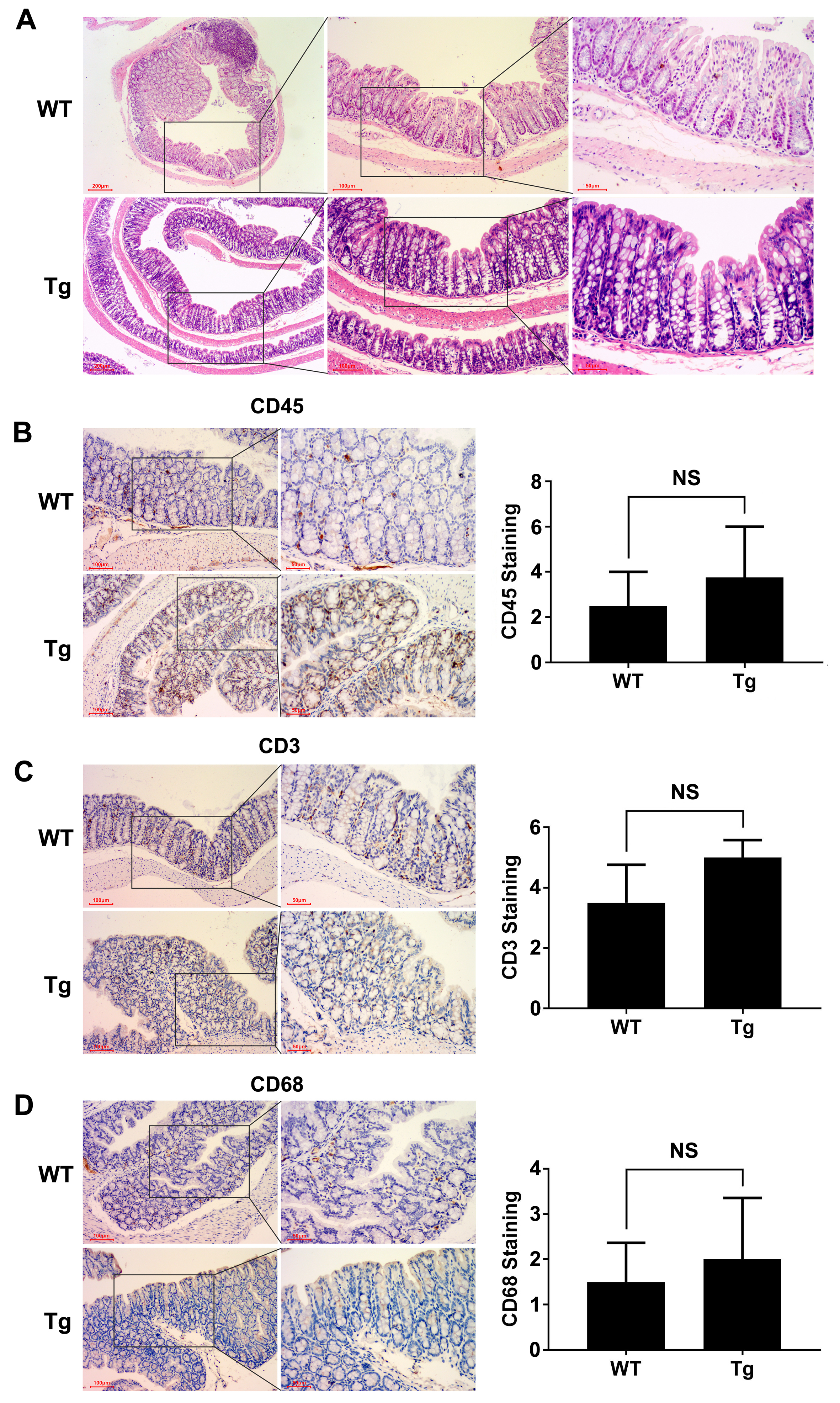

Supplement: Supplementary file 1 — Fig S1 [file JCMM-25-2679-s001.jpg]
