# Supplementary material for: Transgenic overexpression of ITGB6 in intestinal epithelial cells exacerbates dextran sulfate sodium‐induced colitis in mice
Source: J Cell Mol Med. 2021 Jan 24;25(5):2679–90. doi: 10.1111/jcmm.16297 (PMC7933932; doi:10.1111/jcmm.16297)
Supplement: Supplementary file 2 — Supplementary Material [file JCMM-25-2679-s002.docx]

**Supplementary Materials and Methods**

**Western blotting (WB)**

Cells were lysed using fresh and ice-cold protein lysis buffer supplemented with protease inhibitor cocktail (Sigma, St. Louis, MO, USA). Equal amounts of proteins (30 μg) were resolved by sodium dodecyl sulfate-polyacrylamide gel electrophoresis (SDS-PAGE) and then transferred to polyvinylidene difluoride (PVDF) membranes (Bio-Rad, CA, USA). Following blocking, the membranes were incubated with primary antibodies against p-Stat1 (1:500, Cell Signaling, MA, USA), p-Stat5 (1:500, Cell Signaling, MA, USA), p-JNK (1:1000, Cell Signaling, MA, USA), p-ERK (1:500, Cell Signaling, MA, USA), αvβ6 (1:500, ProteinTech, IL, USA), JNK (1:1000, Huabio, CHN), Stat5 (1:1000, Abcam, MA, USA), Stat1(1:1000, Huabio, CHN), ERK (1:10000, Abcam, MA, USA), TGFβ (1:1000, Abcam, MA, USA), fibronectin (1:5000, Abcam, MA, USA), fibrinogen α chain (1:5000, Abcam, MA, USA), fibrinogen β chain (1:5000, Abcam, MA, USA), Gapdh (1:5000, Huabio, CHN) and β-tubulin (1:1000, Cell Signaling, MA, USA), at 4 °C overnight. The blots were then incubated with the relevant secondary antibodies (1:5000, Cell Signaling, MA, USA) for 1 hour at room temperature. Gapdh and β-tubulin was used as an internal control. All experiments were performed in triplicate.

**WB quantification**

To relatively quantify the expression of phosphorylated protein, total protein was used as the loading control by using Image J software (NIH Image, MD, USA) accordingly. The quantification reflected the relative expression as a ratio of each phosphorylated protein relative to the total loading control.

**Immunohistochemistry (IHC) analysis**

Formalin-fixed paraffin-embedded sections were deparaffinized with xylene and rehydrated using a gradient series of alcohol. Following blocking, heat-induced antigen retrieval was performed. The slides were then stained with primary antibodies against CD45 (1:200, Abcam, MA, USA), CD3 (1:200, Abcam, MA, USA), CD68 (1:200, Abcam, MA, USA) and αvβ6 (1:100, Atlas Antibodies, Bromma, Sweden). For evaluation of immunostaining, the immunoreactive score was determined by multiplication of staining intensity and the percentage of positively stained cells (range from 0 to 12). The staining intensity was graded as follows: 0, absent; 1, weak; 2, moderate; and 3, strong. The percentage positivity was ranked as follows: 0, 0%; 1, 1-10%; 2, 11-50%; 3, 51-80%; 4, >80%.

**Supplementary Table 1. Primers for genotyping in generation of Villin1-ITGB6 transgenic mice**

| Name |  | Sequences 5′-3′ |
| --- | --- | --- |
| Vil-GT-tF1 | F | 5′-CAGTCTGAAATAGTGTAGCCTG-3′ |
| Vil-GT-tR1 | R | 5′-ACCTTGTACGTGATCATTCCT-3′ |
| Vil-GT-tF2 | F | 5′-GTGCTGGGCTGTGTAACAGG-3′ |
| Vil-GT-tR2 | R | 5′-CAGGCAGTCTTCACAGGTTT-3′ |
| Vil-GT-tF3 | F | 5′-ATGAAGAAGATGACCGACAAC-3′ |
| Vil-GT-tR3 | R | 5′-ATTTGCCCTCCCATATGTCC-3′ |
| Vil-GT-tF4 | F | 5′-GTACCACGAGTCCAAGTTCT-3′ |
| Vil-GT-tR4 | R | 5′-GCTCAAGGGGCTTCATGATGTC-3′ |

**Supplementary Table 2. Evaluation of disease activity index (DAI)**

| Score | Weight loss (%) | Stool consistency | Occult/Gross bleeding |
| --- | --- | --- | --- |
| 0 | No loss | Normal | Negative |
| 1 | 1-5 | Loose | Occult |
| 2 | 6-10 | Loose | Occult |
| 3 | 11-15 | Loose | Occult |
| 4 | >15 | Diarrhea | Gross bleeding |

**Supplementary Table 3. Primers for quantitative real-time PCR**

| Target mRNA |  | Sequences 5′-3′ |
| --- | --- | --- |
| Homo ITGB6 | F | 5′-TCTGGAGTTGGCGAAAGG-3′ |
|  | R | 5′-TCCACCGGGTAGTCCTCA-3′ |
| Mus IL-1α | F | 5′-CAAGATGGCCAAAGTTCGTGAC-3′ |
|  | R | 5′-GTCTCATGAAGTGAGCCATAGC-3′ |
| Mus IL-1β | F | 5′-ATGGCAACTGTTCCTGAACTCAACT-3′ |
|  | R | 5′-CAGGACAGGTATAGATTCTTTCCTTT-3′ |
| Homo GAPDH | F | 5′-GGAGCGAGATCCCTCCAAAAT-3′ |
|  | R | 5′-GGCTGTTGTCATACTTCTCATGG-3′ |
| Mus Gapdh | F | 5′-GCCCTTGAGCTAGGACTGGA-3′ |
|  | R | 5′-TTTTGTCTACGGGACGAGGC-3′ |
| Mus IL-18 | F | 5′- ACTGTACAACCGCAGTAATACGG -3′ |
|  | R | 5′- AGTGAACATTACAGATTTATCCC -3′ |
| Mus IL-33 | F | 5′- TGAGACTCCGTTCTGGCCTC -3′ |
|  | R | 5′- CTCTTCATGCTTGGTACCCGAT -3′ |
| Mus St2 | F | 5′- ACGCTCGACTTATCCTGTGG-3′ |
|  | R | 5′- CAGGTCAATTGTTGGACACG-3′ |
| Mus IL-6 | F | 5′-GAGGATACCACTCCCAACAGACC-3′ |
|  | R | 5′-AAGTGCATCATCGTTGTTCATACA-3′ |
| Mus IL-10 | F | 5′-ATTTGAATTCCCTGGGTGAGAAG-3′ |
|  | R | 5′-CACAGGGGAGAAATCGATGACA-3′ |
| Mus TNF-α | F | 5′-CCCTCACACTCAGATCATCTTCT-3′ |
|  | R | 5′-GCTACGACGTGGGCTACAG-3′ |
| Mus Fibronectin | F | 5′-ATGTGGACCCCTCCTGATAGT-3′ |
|  | R | 5′-GCCCAGTGATTTCAGCAAAGG-3′ |
| Mus Fibrinogen α chain | F | 5′-AGTCTGGACTACAGATACCGAAG-3′ |
|  | R | 5′-CGTCAATCAACCCTTTCATCCTG-3′ |
| Mus Fibrinogen β chain | F | 5′-ACGATGAACCGACGGATAGC-3′ |
|  | R | 5′-CCGTAGGACACAACACTCCC-3′ |
| Mus Fibrinogen γ chain | F | 5′-ACCAGAGATAACTGTTGCATCCT-3′ |
| Mus Itga3  Mus Itgb4  Mus Itgb5  Mus TGF-β | R  F  R  F  R  F  R  F  R | 5′-CCACGTCGGTTTGGTAAGAAG-3′  5'-CCTCTTCGGCTACTCGGTC-3'  5’-CCGGTTGGTATAGTCATCACCC-3'  5'-GCAGACGAAGTTCCGACAG-3'  5'-GGCCACCTTCAGTTCATGGA-3'  5'-GAAGTGCCACCTCGTGTGAA-3'  5'-GGACCGTGGATTGCCAAAGT-3'  5'-CCACCTGCAAGACCATCGAC-3'  5'-CTGGCGAGCCTTAGTTTGGAC-3' |

**Supplementary Table 4. Characteristics of included IBD patients in our center (ZJU)**

| Patient ID | Gender | Age at Diagnosis | Diagnosis | Specimen Location | Treatment | | Disease Activity |
| --- | --- | --- | --- | --- | --- | --- | --- |
| 1 | Female | 32 | CD | Small intestine | 5-aminosalicylate therapy | Active | |
| 2 | Male | 23 | CD | Large intestine | Azathioprine therapy | Active | |
| 3 | Male | 34 | CD | Small intestine | Azathioprine therapy | Active | |
| 4 | Female | 32 | CD | Large intestine | Azathioprine therapy | Active | |
| 5 | Male | 31 | CD | Small intestine | Azathioprine therapy | Active | |
| 6 | Female | 33 | CD | Large intestine | Azathioprine and corticosteroid therapy | Active | |
| 7 | Male | 25 | CD | Large intestine | None | Active | |
| 8 | Female | 33 | UC | Large intestine | Corticosteroid therapy | Active | |
| 9 | Male | 35 | CD | Small intestine | 5-aminosalicylate and corticosteroid therapy | Active | |
| 10 | Male | 31 | CD | Small intestine | None | Active | |
| 11 | Male | 34 | CD | Small intestine | Azathioprine and corticosteroid therapy | Active | |
| 12 | Female | 40 | CD | Large intestine | Thalidomide therapy | Active | |
| 13 | Female | 38 | CD | Small intestine | None | Active | |
| 14 | Female | 26 | CD | Small intestine | None | Active | |
| 15 | Female | 19 | CD | Small intestine | Corticosteroid therapy | Active | |
| 16 | Female | 37 | CD | Small intestine | Azathioprine and corticosteroid therapy | Active | |
| 17 | Male | 26 | CD | Small intestine | Azathioprine therapy | Active | |

**Supplementary Table 5-1. Characteristics of GSE11223 and GSE20881 datasets**

| Dataset | Total number | | Diagnosis | Sample | Country | First author  (publication year) |
| --- | --- | --- | --- | --- | --- | --- |
|  | Normal | IBD |  |  |  |  |
| GSE11223 | 135 | 67 | UC | Mucosa | UK | Noble et al (2008) |
| GSE20881* | 73 | 99 | CD | Mucosa | UK | Noble et al (2010) |

*number of biopsies. 73 biopsies from 31 healthy controls and 99 biopsies from CD.

**Supplementary Table 5- 2. Characteristics of GSE38713 dataset**

| Dataset | Total number | | Diagnosis | Sample | Country | First author (publication year) |
| --- | --- | --- | --- | --- | --- | --- |
|  | Remission | Active |  |  |  |  |
| GSE38713 | 8 | 15 | UC | Mucosa | Spain | Planell et al (2013) |

**Supplementary Table 6. Differentially expressed cytokines between WT and TG mice**

| Symbol | Well | AVG ΔCt  (TG) | AVG ΔCt  (WT) | 2^-ΔCt  (TG) | 2^-ΔCt  (WT) | Fold Change |
| --- | --- | --- | --- | --- | --- | --- |
| Cxcl5 | C12 | 15.164 | 12.932 | 2.72384E-05 | 0.000127962 | 0.212863427 |
| Gusb | H04 | 6.474 | 4.962 | 0.011249463 | 0.032084048 | 0.350624813 |
| Il18 | D11 | 11.784 | 13.652 | 0.000283571 | 7.76852E-05 | 3.650261954 |
| Ccl20 | A11 | 9.184 | 8.392 | 0.001719256 | 0.002976845 | 0.577542892 |
| Cxcr2 | D03 | 13.814 | 13.502 | 6.94339E-05 | 8.61972E-05 | 0.805524291 |
| Tlr4 | G05 | 5.434 | 5.122 | 0.023131458 | 0.028716028 | 0.805524291 |
| Cxcl1 | C07 | 15.164 | 14.952 | 2.72384E-05 | 3.155E-05 | 0.863339559 |
| Cxcr4 | D04 | 6.514 | 6.342 | 0.010941846 | 0.012327294 | 0.887611337 |
| Il1b | E01 | 12.264 | 12.102 | 0.000203314 | 0.000227476 | 0.893785162 |
| Fos | D06 | 0.744 | 0.592 | 0.597081594 | 0.66342257 | 0.90000193 |
| Cxcl2 | C10 | 12.134 | 11.992 | 0.000222486 | 0.000245498 | 0.906261938 |
| Crp | C05 | 14.934 | 14.872 | 3.19461E-05 | 3.33489E-05 | 0.957935218 |
| Cxcl3 | C11 | 13.894 | 13.932 | 6.56885E-05 | 6.39809E-05 | 1.026689546 |
| Cd14 | C01 | 3.734 | 4.012 | 0.075154328 | 0.061982296 | 1.212512819 |
| Nos2 | F08 | 6.254 | 6.612 | 0.013102628 | 0.010223266 | 1.281647924 |
| Tlr7 | G08 | 9.714 | 10.102 | 0.001190679 | 0.000909903 | 1.308578071 |
| Ptgs2 | F10 | 8.614 | 9.012 | 0.002552276 | 0.001936947 | 1.317679952 |
| Tnf | G10 | 7.164 | 7.632 | 0.006973023 | 0.00504126 | 1.383190629 |
| Hsp90ab1 | H05 | -0.646 | -0.098 | 1.564823563 | 1.070288698 | 1.462057448 |
| Il6 | E09 | 15.074 | 15.652 | 2.89917E-05 | 1.94213E-05 | 1.492778383 |
| Il1rn | E04 | 5.724 | 6.392 | 0.018919267 | 0.011907381 | 1.5888688 |
| Tlr1 | G02 | 3.114 | 3.872 | 0.115502822 | 0.068298609 | 1.691144575 |
| Il7 | E11 | 7.394 | 8.392 | 0.005945443 | 0.002976845 | 1.997229332 |
| Tirap | G01 | 5.834 | 6.852 | 0.017530367 | 0.008656503 | 2.025109615 |
| Ccl1 | A05 | 15.164 | 16.252 | 2.72384E-05 | 1.28133E-05 | 2.125791349 |
| Cd40lg | C03 | 15.164 | 16.252 | 2.72384E-05 | 1.28133E-05 | 2.125791349 |
| Cxcr1 | D02 | 15.164 | 16.252 | 2.72384E-05 | 1.28133E-05 | 2.125791349 |
| Il17a | D10 | 15.164 | 16.252 | 2.72384E-05 | 1.28133E-05 | 2.125791349 |
| Il9 | E12 | 15.164 | 16.252 | 2.72384E-05 | 1.28133E-05 | 2.125791349 |
| Tlr5 | G06 | 3.684 | 4.772 | 0.07780464 | 0.036600318 | 2.125791349 |
| C4b | A04 | 6.604 | 7.802 | 0.010280113 | 0.004480887 | 2.294214048 |
| C3ar1 | A03 | 9.974 | 11.212 | 0.000994321 | 0.000421553 | 2.358713185 |
| Bcl6 | A01 | 8.524 | 9.872 | 0.002716566 | 0.001067166 | 2.545589871 |
| Ccr7 | B12 | 11.154 | 12.552 | 0.000438845 | 0.000166522 | 2.635359903 |
| Ccr2 | B09 | 6.894 | 8.352 | 0.008408126 | 0.003060536 | 2.747272467 |
| Cd40 | C02 | 10.514 | 12.072 | 0.000683865 | 0.000232255 | 2.944453724 |
| Tlr3 | G04 | 7.224 | 8.792 | 0.006688971 | 0.002256027 | 2.96493402 |
| C3 | A02 | 3.984 | 5.562 | 0.063197005 | 0.021167578 | 2.985556767 |
| Ccl3 | B03 | 10.324 | 11.932 | 0.000780129 | 0.000255923 | 3.048289661 |
| Ccr3 | B10 | 9.084 | 10.712 | 0.001842653 | 0.000596165 | 3.090842199 |
| Tlr6 | G07 | 7.784 | 9.492 | 0.004537143 | 0.001388747 | 3.267075964 |
| Ccl22 | A12 | 8.084 | 9.812 | 0.003685306 | 0.001112484 | 3.312682645 |
| Il6ra | E10 | 8.224 | 9.952 | 0.003344485 | 0.0010096 | 3.312682645 |
| Tollip | G12 | 3.194 | 4.932 | 0.109272328 | 0.032758202 | 3.335724175 |
| Csf1 | C06 | 7.094 | 8.842 | 0.007319699 | 0.002179178 | 3.358925972 |
| Ccl7 | B06 | 9.674 | 11.462 | 0.001224153 | 0.000354482 | 3.453358234 |
| Tlr9 | G09 | 9.554 | 11.352 | 0.00133033 | 0.000382567 | 3.47737824 |
| Il10 | D08 | 3.224 | 2.412 | 0.107023536 | 0.187895185 | 0.569591689 |
| Ccl12 | A07 | 8.484 | 10.462 | 0.002792939 | 0.000708964 | 3.939465772 |
| Tnfsf14 | G11 | 10.184 | 12.162 | 0.000859628 | 0.000218209 | 3.939465772 |
| Fasl | D05 | 12.234 | 14.262 | 0.000207586 | 5.08991E-05 | 4.078390732 |
| Il1a | D12 | 10.284 | 12.442 | 0.000802061 | 0.000179715 | 4.462957289 |
| Ccr1 | B08 | 7.994 | 10.152 | 0.003922529 | 0.000878908 | 4.462957289 |
| Ifng | D07 | 11.774 | 13.962 | 0.000285544 | 6.26642E-05 | 4.556733509 |
| Ccl19 | A09 | 8.464 | 10.662 | 0.002831927 | 0.000617189 | 4.588428097 |
| Ccl5 | B05 | 4.334 | 6.632 | 0.049583365 | 0.010082519 | 4.917755469 |
| Sele | F12 | 11.844 | 14.162 | 0.00027202 | 5.45523E-05 | 4.986404777 |
| Ccr4 | B11 | 9.454 | 11.842 | 0.001425813 | 0.000272397 | 5.234312286 |
| Ccl2 | A10 | 7.914 | 10.312 | 0.004146184 | 0.000786645 | 5.270719807 |
| Il10rb | D09 | 0.604 | 3.092 | 0.657927263 | 0.11727765 | 5.609997003 |
| Ccl4 | B04 | 8.394 | 11.012 | 0.002972721 | 0.000484237 | 6.138984384 |
| Ccl8 | B07 | 2.184 | 4.832 | 0.220064753 | 0.035109372 | 6.267977496 |
| Il22 | E05 | 12.444 | 15.112 | 0.000179466 | 2.82381E-05 | 6.355475201 |
| Ccl25 | B02 | 6.134 | 8.812 | 0.014239083 | 0.002224968 | 6.399681027 |
| Ltb | F04 | 5.504 | 8.242 | 0.022035906 | 0.003303017 | 6.671448351 |
| Ly96 | F05 | 6.424 | 9.262 | 0.011646175 | 0.001628772 | 7.150281299 |
| Itgb2 | F01 | 7.454 | 10.322 | 0.00570325 | 0.000781211 | 7.300523908 |
| Cebpb | C04 | 6.404 | 9.352 | 0.011808749 | 0.001530268 | 7.716785477 |
| Il1r1 | E02 | 4.304 | 7.262 | 0.050625217 | 0.006515086 | 7.770459964 |
| Tlr2 | G03 | 3.994 | 6.972 | 0.062760471 | 0.007965607 | 7.878931543 |
| Ccl17 | A08 | 7.774 | 10.952 | 0.004568702 | 0.0005048 | 9.050515703 |
| Cxcl11 | C09 | 9.984 | 13.282 | 0.000987453 | 0.000100397 | 9.835510938 |
| Il1rap | E03 | 5.254 | 8.662 | 0.026205256 | 0.002468756 | 10.61476112 |
| Myd88 | F06 | 5.664 | 9.132 | 0.019722688 | 0.001782354 | 11.06552503 |
| Lta | F03 | 9.684 | 13.272 | 0.001215697 | 0.000101095 | 12.02529182 |
| Ripk2 | F11 | 5.764 | 9.432 | 0.018401919 | 0.001447722 | 12.7109504 |
| Ccl24 | B01 | 12.554 | 16.252 | 0.000166291 | 1.28133E-05 | 12.97803449 |
| Nfkb1 | F07 | 0.424 | 4.242 | 0.745355193 | 0.052848268 | 14.10368253 |
| Cxcl10 | C08 | 5.884 | 9.842 | 0.016933218 | 0.001089589 | 15.54091993 |
| Cxcl9 | D01 | 12.094 | 16.252 | 0.000228741 | 1.28133E-05 | 17.85182916 |
| Il5 | E08 | 9.494 | 13.702 | 0.001386824 | 7.5039E-05 | 18.48137255 |
| Nr3c1 | F09 | 0.074 | 4.592 | 0.950000383 | 0.041463911 | 22.91149986 |
| Il23r | E07 | 9.994 | 14.562 | 0.000980632 | 4.13429E-05 | 23.71947216 |
| Il23a | E06 | 9.814 | 14.422 | 0.001110943 | 4.5556E-05 | 24.38631729 |

**Supplementary Figure Legend**

Conditional overexpression of ITGB6 in IECs showed no histopathological features difference. A, HE examination of large intestines in wild type (WT) and transgenic (Tg) mice. B-D, The infiltration of CD45^+^ immune cells (B), CD3^+^ T cells (C) and CD68^+^ macrophages (D) in large intestines were determined by IHC.
